# L2_1_ and XA Ordering Competition in Hafnium-Based Full-Heusler Alloys Hf_2_VZ (Z = Al, Ga, In, Tl, Si, Ge, Sn, Pb)

**DOI:** 10.3390/ma10101200

**Published:** 2017-10-20

**Authors:** Xiaotian Wang, Zhenxiang Cheng, Wenhong Wang

**Affiliations:** 1 School of Physical Science and Technology, Southwest University, Chongqing 400715, China; wangxt45@126.com or xiaotianwang@swu.edu.cn; 2 Institute for Superconducting and Electronic Materials, University of Wollongong, Wollongong 2500, Australia; 3 Beijing National Laboratory for Condensed Matter Physics, Institute of Physics, Chinese Academy of Sciences, Beijing 100190, China; wenhong.wang@iphy.ac.cn

**Keywords:** site preference, Hf-based full-Heusler compounds, first-principles study, band structures, magnetic properties, mechanical properties

## Abstract

For theoretical designing of full-Heusler based spintroinc materials, people have long believed in the so-called Site Preference Rule (SPR). Very recently, according to the SPR, there are several studies on XA-type Hafnium-based Heusler alloys X_2_YZ, i.e., Hf_2_VAl, Hf_2_CoZ (Z = Ga, In) and Hf_2_CrZ (Z = Al, Ga, In). In this work, a series of Hf_2_-based Heusler alloys, Hf_2_VZ (Z = Al, Ga, In, Tl, Si, Ge, Sn, Pb), were selected as targets to study the site preferences of their atoms by first-principle calculations. It has been found that all of them are likely to exhibit the L2_1_-type structure instead of the XA one. Furthermore, we reveal that the high values of spin-polarization of XA-type Hf_2_VZ (Z = Al, Ga, In, Tl, Si, Ge, Sn, Pb) alloys have dropped dramatically when they form the L2_1_-type structure. Also, we prove that the electronic, magnetic, and physics nature of these alloys are quite different, depending on the L2_1_-type or XA-type structures.

## 1. Introduction

Heusler alloys are a noticeable class of intermetallic materials that represent as usual by the formula X_2_YZ (often called full-Heusler) [1,2,3,4,5,6,7,8,9,10,11,12,13,14,15] or XYZ (usually named as half-Heusler) [16], where X, Y are transition-metal-element atoms and Z is a main group element. The structure of full-Heusler alloys consists of four interpenetrating fcc lattices with four equidistant sites as basis along the diagonal of the unit cell. According to the well-known Site Preference Rule (SPR) [1,2,3,4,5,6,7,8,9,10,11,12,13,14,15], when the valence of the X is larger than that of Y, the atomic sequence is X^A^-Y^B^-X^C^-Z^D^ and the structure is the well-known L2_1_ one with prototype Cu_2_MnAl. Otherwise, the alloys crystallize in the so-called XA structure, where the sequence of the atoms is then X^A^-X^B^-Y^C^-Z^D^ and the prototype is Hg_2_CuTi. The latter alloys are also named as inverse Heusler alloys.

To the best of our knowledge, SPR has been applied extensively in the theoretical design of full-Heusler alloys and in predictions of their electronic, magnetic, and transport behavior. Some XA-type full-Heusler alloys, Mn_2_CoAl [16], Ti_2_MnAl [17], and Ti_2_CoSi [18], were predicted to be novel spin-gapless semiconductors (SGSs) [19,20]. Furthermore, lots of XA-type full-Heusler alloys, Sc_2_-, V_2_-, Cr_2_-, Mn_2_-, Ti_2_-, Zr_2_-, and even Hf_2_-based alloys, were revealed to be excellent half-metallic materials (HMMs) [21]. Surprisingly, one counterexample after another, including X_2_CuAl [22] and Ti_2_FeZ (Z = Al, Ga) alloys [6], has been reported very recently, in which these alloys show the L2_1_-type structure and disobey the SPR, so that Y with more electrons enters the B sites. 

In this work, a systematic theoretical work has been carried out to examine whether the conventional SPR was suitable for the Hf_2_-based highly-ordered full-Heusler alloys. To this end, the competition between the XA and L2_1_ orderings of Hf_2_VZ (Z = Al, Ga, In, Tl, Si, Ge, Sn, Pb) full-Heusler alloys has been studied through the first-principles calculations. Our current work shows that not all the full-Heusler alloys obey the SPR, and further, the SPR can not be considered as a concise judgement principle for the structure of highly-ordered full-Heusler alloys. Remarkably, we exhibit that atomic site occupation in these Hf_2_VZ alloys is decisive in determining their electronic, magnetic, and Slater-Pauling properties. For the L2_1_-type Hf_2_VZ (Z = Al, Ga, In, Tl, Si, Ge, Sn, Pb) full-Heusler alloys, the phase stability from the aspect of the formation energy and mechanical behaviors has also been examined in term of theory. Details of the results are shown in the following discussion.

## 2. Computational Details

First-principles electronic-structure calculations were performed using density function theory (DFT) implemented in the CASTEP code [23,24] according to the plane-wave pseudo-potential method. The generalized gradient approximation (GGA) [25] was adopted for the exchange-correlation functional. For the XA-type and L2_1_-type Heusler alloys Hf_2_VZ (Z = Al, Ga, In, Tl, Si, Ge, Sn, Pb), a Monkhorst-Pack special k-point mesh of 9 × 9 × 9 was used in the Brillouin zone integrations with a cutoff energy of 400 eV and a self-consistent field tolerance of 10^−6^ eV. The quality of the k-point separation for the band structure calculation is 0.01 Å^−1^. 

**Figure 1:**
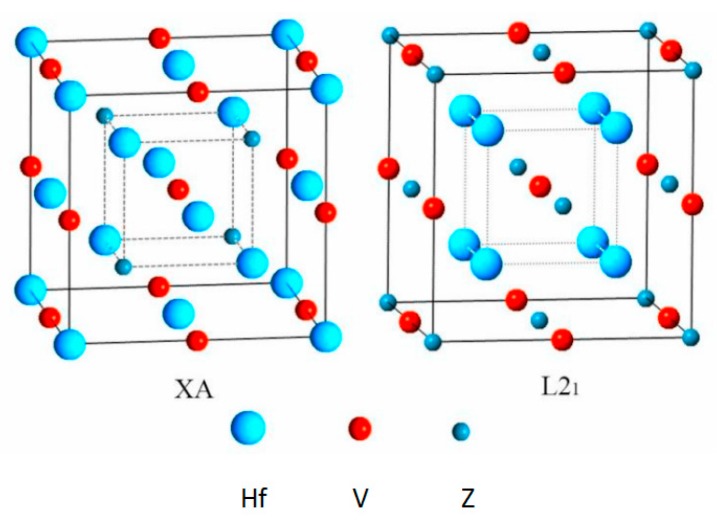
Crystal structures of XA and L2_1_ types Hf_2_VZ (Z = Al, Ga, In, Tl, Si, Ge, Sn, Pb) alloys. This 1 × 1 × 1 super-cell system contains 16 atoms, i.e., 8 × Hf, 4 × V and 4 × Z.

As shown in Figure 1, the crystal structures of XA and L2_1_ types Hf_2_VZ Heusler alloys have been given. For the former, the two Hf atoms sitting at the two inequivalent sites, and Hf 1, Hf 2, V and Z atoms occupy the Wyckoff coordinates A (0, 0, 0), B (0.25, 0.25, 0.25), C (0.5, 0.5, 0.5) and D (0.75, 0.75, 0.75), respectively; For the latter, the two Hf atoms are essentially equivalent, and they have the same atomic environment. Hf 1, Hf 2, V and Z atoms occupy the Wyckoff coordinates A (0, 0, 0), B (0.5, 0.5, 0.5), C (0.25, 0.25, 0.25) and D (0.75, 0.75, 0.75), respectively.

## 3. Results and Discussion

### 3.1. Competition of L2_1_ and XA Structure Ordering in Full-Heusler Hf_2_VZ Alloys

First, to confirm the ground state of Hf_2_VZ (Z = Al, Ga, In, Tl, Si, Ge, Sn, Pb) full-Heusler alloys, the geometry optimization has been performed by calculating the total energies as functions of the lattice constant (cell volume) [26]. In this work, three possible magnetic states, i.e., paramagnetic (PM), ferrimangetic (FiM), and antiferromagnetic (AFM) states are taken into account. The PM (or nonmagnetic) state means that the constituent atoms of Hf_2_VZ have no spin polarization. The FiM state implies that the spin magnetic moments of Hf atoms align anti-parallel to those of the V atoms, and the total magnetic moment is not equal to zero, while the AFM state means the spin magnetic moments of Hf atoms align antiparallelly to those of the V atoms, and the total magnetic moment is equal to zero. For our Hf_2_VZ (Z = Al, Ga, In, Tl, Si, Ge, Sn, Pb) full-Heusler alloys, the FiM state is the most stable among three magnetic states. 

**Figure 2:**
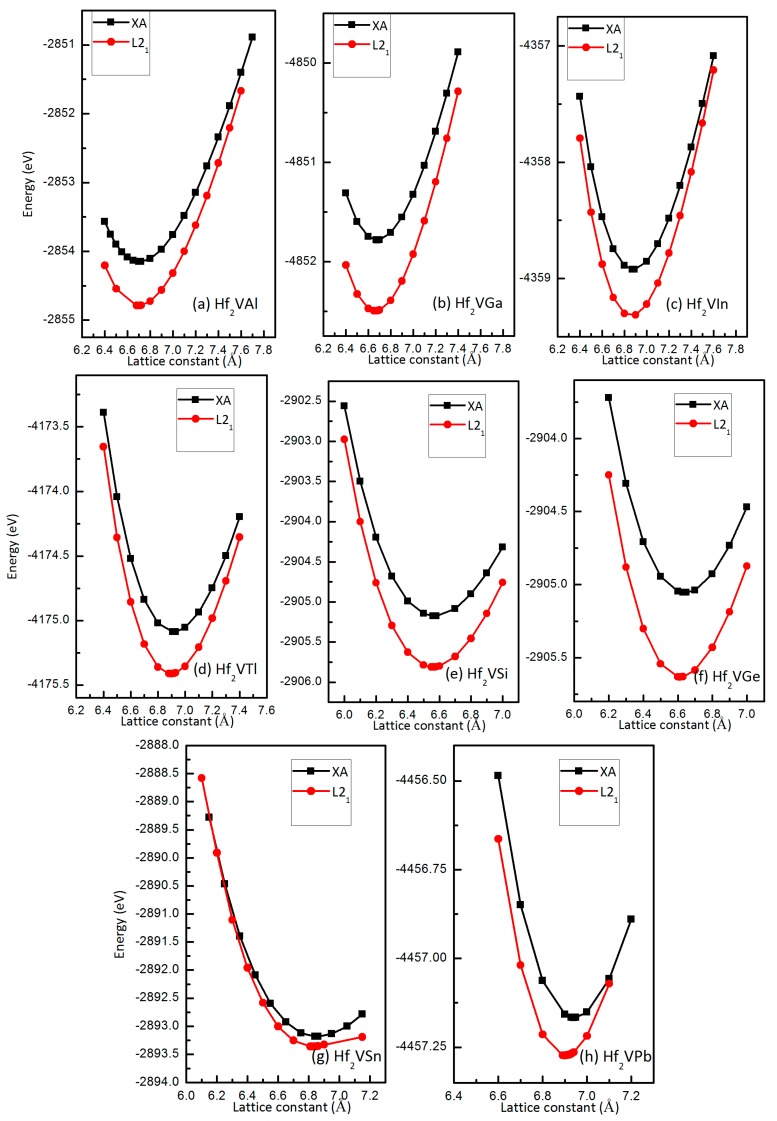
Calculated total energies of XA and L2_1_ types Hf_2_VAl (**a**); Hf_2_VGa (**b**); Hf_2_VIn (**c**); Hf_2_VTl (**d**); Hf_2_VSi (**e**); Hf_2_VGe (**f**); Hf_2_VSn (**g**); Hf_2_VPb (**h**) alloys with respect to the lattice constant.

As shown in Figure 2, the total energy as functions of lattice constants of full-Heusler Hf_2_VZ (Z = Al, Ga, In, Tl, Si, Ge, Sn, Pb) alloys with two atomic occupation orderings, XA and L2_1_, and with their most stable magnetic state, FiM, have been exhibited. Obviously, for all these alloys, the L2_1_ state has lower energies than XA state. Therefore, Hf_2_VZ alloys studied in current work prefer to form the L2_1_-type structure as ground state with equilibrium lattice constant of 6.69 Å, 6.66 Å, 6.90 Å, 6.89 Å, 6.56 Å, 6.61 Å, 6.82 Å and 6.90 Å, respectively. The obtained equilibrium lattice constants of these alloys with XA-type and L2_1_-type structures, respectively, and the energy differences between these two structures ($\Delta E=E_{XA}^{total}-E_{L2_{1}}^{total}$) for Hf_2_VZ (Z = Al, Ga, In, Tl, Si, Ge, Sn, Pb) alloys are also listed in Table 1. A higher value of *ΔE* indicates the L2_1_-type structure is more stable than XA-type. The highest positive value of 0.71 eV/cell appears in Hf_2_VGa alloy, reflecting that the site preference of V for the B position is quite strong. Hence, compared to other Hf_2_-based Heusler alloys, XA-type Hf_2_VGa maybe more difficult to synthesize experimentally due to its largest *ΔE*.

We should point out here that, as we said in the part of Introduction, Hf_2_VZ alloys or even all the Hf-based full Heusler alloys should exhibit XA-type Heusler structure on basis of the SPR. Surprisingly, in current work, our results break the traditional SPR. Also, the data in this work, together with the latest scientific findings in References [6,22,27,28], are sufficient to demonstrate that not all the full-Heusler alloys X_2_YZ obey the well-known SPR, especially X are low-valent transition metals. Since the SPR can not be regarded as the only way to determine the competition of XA and L2_1_ structural ordering in Heusler alloys, alloys with L2_1_-type structure should also be taken into account in the previous works [1,2,3,7,8,9,10,11].

### 3.2. Structural and Mechanical Properties of Heusler-Based Hf_2_VZ with L2_1_-Type Ordering

Further, we aim to check the structural stability of the L2_1_-type full-Heusler Hf_2_VZ according to their calculated formation energies and mechanical properties. Similar methods [29,30] have been applied extensively to analyze the stability for Heusler alloys in term of theory. The formation energies of these alloys can be obtained from the following equation:

(1) $$E_{f}=E_{Hf_{2}VZ}^{total}-(E_{Hf1}^{bulk}+E_{Hf2}^{bulk}+E_{V}^{bulk}+E_{Z}^{bulk})$$

where $E_{Hf_{2}VZ}^{total}$ is the total energy of Hf_2_VZ per formula unit, and $E_{Hf1}^{bulk}$, $E_{Hf2}^{bulk}$, $E_{V}^{bulk}$, and $E_{Z}^{bulk}$ are the total energies per atom of each element in the bulk form for the Hf 1, Hf 2, V, and Z, respectively. The results have been given in Table 2, we can see that all these alloys have negative formation energies. Furthermore, the total energy of L2_1_-type Hf_2_VZ is lower than XA-type Hf_2_VZ (see Figure 1), and thus, the *E_f_* of L2_1_-type Hf_2_VZ should be lower than XA-type Hf_2_VZ. This implies that L2_1_-type Hf_2_VZ should be more stable than the XA-type Hf_2_VZ alloys.

Next, we come to the mechanical properties of these alloys and examine their stability based on achieved elastic constants C*_ij_*. For these alloys with cubic structure, only three independent elastic constants (C_11_, C_12_ and C_44_) are needed to be taken into consideration, and the C*_ij_* can be shown as below [31]:

(2) $$\left(\begin{array}{cccccc} C_{11} & C_{12} & C_{12} & 0 & 0 & 0\\ C_{12} & C_{11} & C_{12} & 0 & 0 & 0\\ C_{12} & C_{12} & C_{11} & 0 & 0 & 0\\ 0 & 0 & 0 & C_{44} & 0 & 0\\ 0 & 0 & 0 & 0 & C_{44} & 0\\ 0 & 0 & 0 & 0 & 0 & C_{44} \end{array}\right)$$

For a small strain on a cubic system, the change of elastic energy *ΔE* can be shown as below [32]:

(3) $$\Delta E=\frac{V}{2}\sum_{i=1}^{6}\sum_{i=1}^{6}C_{ij}e_{i}e_{j}$$

where *V* stands for the volume of the unit cell. The strain tensors are always symmetric, and they can therefore be expressed more compactly as 6-component vectors, using the so-called Voigt notation. We select three strain tensors (0,0,0,δ,δ,δ), (δ,δ,0,0,0,0) and (δ,δ,δ,0,0,0) to obtain the elastic constants:

(4) $$\frac{\Delta E_{1}}{V}=\frac{3}{2}C_{44}\delta^{2}$$

(5) $$\frac{\Delta E_{2}}{V}=(C_{11}+C_{12})\delta^{2}$$

(6) $$\frac{\Delta E_{3}}{V}=\frac{3}{2}(C_{11}+C_{12})\delta^{2}$$

where $\Delta E_{1},\Delta E_{2},\Delta E_{3}$ are the change of elastic energy for small strain tensors (0,0,0,δ,δ,δ), (δ,δ,0,0,0,0) and (δ,δ,δ,0,0,0), respectively. 

Based on obtained C*_ij_*, the mechanical properties, including bulk modulus *B*, shear modulus *G*, Voigt’s shear modulus *G_V_*, Reuss’s shear modulus *G_R_*, Young’s modulus *E*, Pugh’s ratio *B*/*G*, anisotropy factor *A*, of Hf_2_VZ can be calculated by using the following equations [29]:

(7) $$B=\frac{C_{11}+2C_{12}}{3}$$

(8) $$G=\frac{G_{R}+G_{V}}{2}$$

(9) $$G_{V}=\frac{C_{11}-C_{12}+3C_{44}}{5}$$

(10) $$G_{R}=\frac{5(C_{11}-C_{12})C_{44}}{4C_{44}+3(C_{11}-C_{12})}$$

(11) $$E=\frac{9GB}{3B+G}$$

(12) $$A=\frac{2C_{44}}{C_{11}-C_{12}}$$

As shown in Table 2, we can see that all these alloys with L2_1_-type structure are mechanical stable due to their calculated elastic constants follow the generalized elastic stability criteria [33]:

(13) $$C_{44}>0$$

(14) $$\frac{\left(C_{11}-C_{12}\right)}{2}>0$$

(15) B>0

(16) $$C_{12}<B<C_{11}$$

Moreover, some special mechanical properties of these alloys can also be observed from Table 2. We addressed some as follows: (1) the values of *B/G* of these alloys are all larger than 1.75, reflecting they are ductile according to Pugh’s criteria; (2) the values of *A* for all these alloys are not equal to 1, meaning the fact that they are anisotropic; (3) as is known, the higher the value of *E*, the stiffer is the materials, and therefore, the relative stiffer order of these Hf_2_VZ materials is Hf_2_VAl > Hf_2_VSi > Hf_2_VGa > Hf_2_VGe > Hf_2_VIn > Hf_2_VSn > Hf_2_VTl > Hf_2_VPb.

### 3.3. Calculated Electronic Behaviors of L2_1_ and XA Types Hf_2_VZ

**Figure 3:**
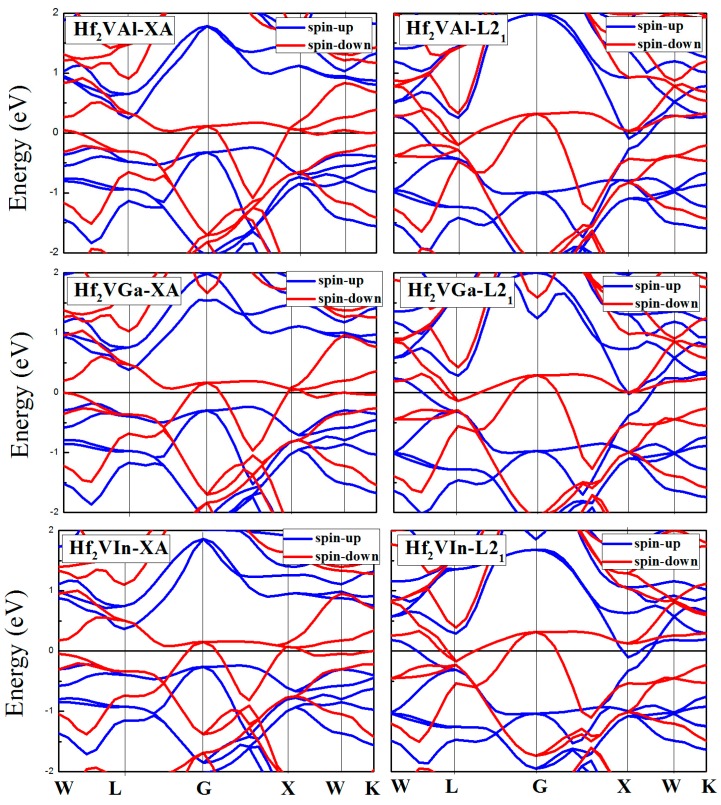
Calculated band structures of XA and L2_1_ types Hf_2_VZ (Z = Al, Ga, In) alloys.

**Figure 4:**
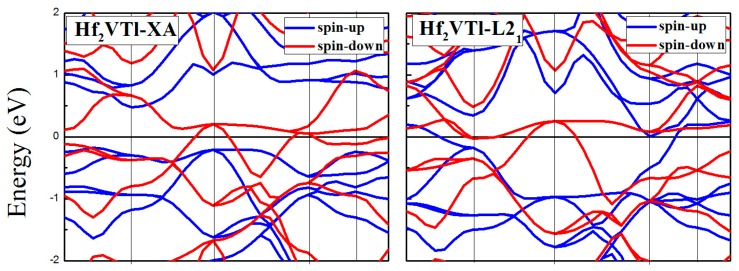

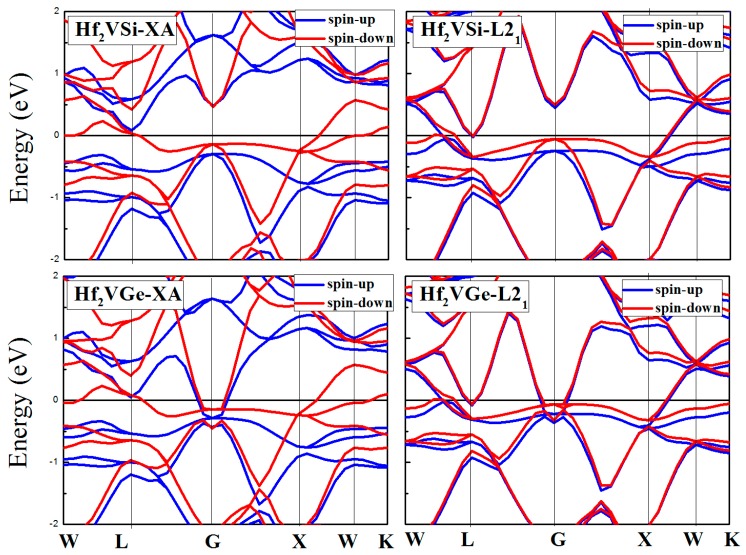
Calculated band structures of XA and L2_1_ types Hf_2_VZ (Z = Tl, Si, Ge) alloys.

**Figure 5:**
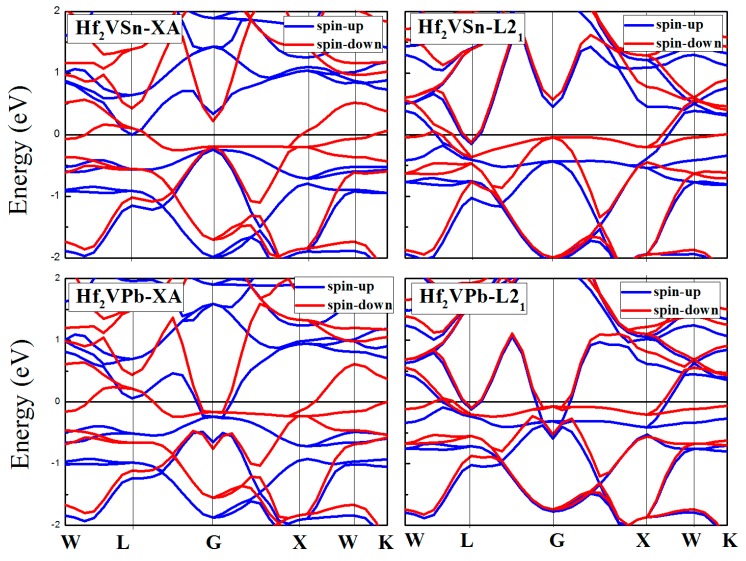
Calculated band structures of XA and L2_1_ types Hf_2_VZ (Z = Sn and Pb) alloys.

Electronic-structure calculation theory has clearly indicated [20,34,35] that the spintonic properties of materials among Heusler family has an unusual sensibility to the atomic occupation in crystal cell. Hence, in this section, a discussion about the spintronic property differences, including the magnetic, electronic, half-metallic, and the spin polarization ratio (*p*), between the XA and L2_1_ Hf_2_VZ (Z = Al, Ga, In, Tl, Si, Ge, Sn, Pb) full-Heusler alloys should be performed. Figure 3, Figure 4 and Figure 5 show the calculated band structures for XA and L2_1_ types Hf_2_VZ (Z = Al, Ga, In, Tl, Si, Ge, Sn, Pb) at their equilibrium lattice constants.

For XA atomic ordering, Hf_2_VZ (Z = Al, Ga, In, Tl, Si, Sn) alloys are excellent half-metallic materials since there is a semiconducting-type band gap in the spin-up direction and the Fermi level locates between the opened gap. However, in the spin-down direction, the semiconducting-type band gap disappeared: although an opened gap can be observed near the Fermi level, the Fermi level has overlapped with the spin-down bands in varying degrees. In the spin-up channel, the significant factors, including the calculated valence band maximum (VBM), conduction band minimum (CBM), semiconducting band gap, and spin-flip/half-metallic gap, have been given in Table 3, the semiconducting band gap [36] is the sum of the absolute values of CBM and VBM, and the spin-flip/half-metallic gap [36] is defined as the minimum value of the two absolute values of CBM and VBM. The semiconducting band gap values of these alloys are 0.46 eV for Hf_2_VAl, 0.59 eV for Hf_2_VGa, 0.59 eV for Hf_2_VIn, 0.64 eV for Hf_2_VTl, 0.35 eV for Hf_2_VSi, 0.30 eV for Hf_2_VSn, respectively. The spin-flip/half-metallic gap values of these alloys are 0.23 eV for Hf_2_VAl, 0.23 eV for Hf_2_VGa, 0.22 eV for Hf_2_VIn, 0.17 eV for Hf_2_VTl, 0.07 eV for Hf_2_VSi, 0.04 eV for Hf_2_VSn, respectively. For XA atom ordering Hf_2_VGe and Hf_2_VPb alloys, semiconducting band gaps in the both directions disappeared and both alloys exhibit common metallic properties.

For Hf_2_VZ (Z = Al, Ga, In, Tl, Si, Sn) alloys with the XA structure, the V and Hf atoms occupy sites with same symmetry in XA-type Heusler structure, and the hybridization of their d orbitals creates 5 bonding bands (3*t*_2g_ and 2*e*_g_) and 5 non-bonding bands (2*e*_u_ and 3*t*_1u_). Then, the 5 V-Hf bonding d hybrids hybridize in turn with d orbitals of Hf, again forming bonding and anti-bonding bands, while the 5 non-bonding bands (2*e*_u_ and 3*t*_1u_) still exist with no hybridizing. Finally, the distribution of the 15 d orbitals in the minority-spin direction can be determined, i.e., 3*t*_2g_, 2*e*_g_, 2*e*_u_, 3*t*_1u_, 3*t*_2g_, and 2*e*_g_, from the high-energy level to the low-energy level. Also, we cannot ignore that Z creates 1s and 3p bands which are totally occupied in Hf_2_VZ and are also below the above-mentioned 15 d orbitals. Therefore, their semiconducting-type band gaps are created by the separated $\Gamma_{15}$ and $\Gamma_{25}$ states, coming from the bonding *t*_2g_ and antibonding *t*_1u_ states.

**Figure 6:**
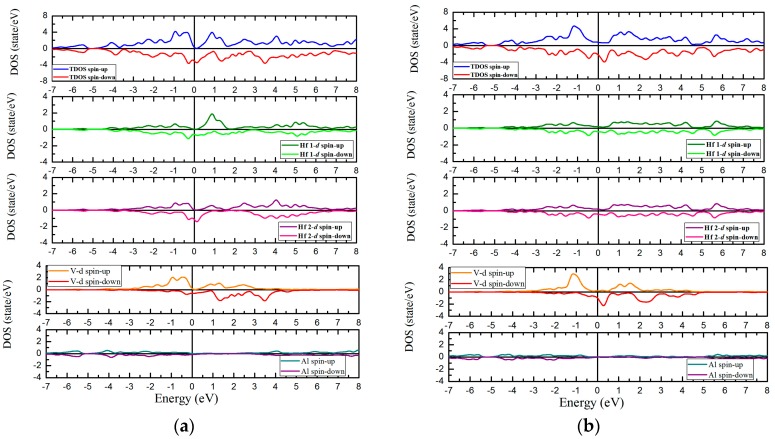

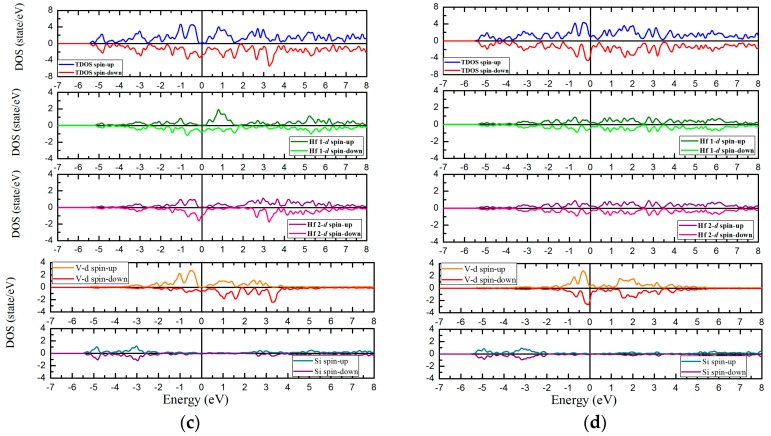
Calculated band structures of XA type Hf_2_VAl (**a**); L2_1_ type Hf_2_VAl (**b**); XA type Hf_2_VSi (**c**); L2_1_ type Hf_2_VSi (**d**); respectively.

The calculated total and partial density of states (TDOS and PDOS) for the six Hf_2_VZ (Z = Al, Ga, In, Tl, Si, Sn) alloys with XA and L2_1_ atomic orderings and for equilibrium lattice constants have also been calculated in this work to deepen the understanding of their electronic properties. As an example, the results of Hf_2_VAl and Hf_2_VSi alloys are given in Figure 6. Clearly, from the figure, a semiconducting band gap can be found in spin-up direction for XA-type Hf_2_VZ (Z = Al, Ga, In, Tl, Si, Sn) alloys, which is in a good agreement with above-mentioned band structures.

The DOS can be widely used to analyze the bonding/anti-bonding states and the gap formation and similar analytical approach can be observed in References [19,29]. In the spin-up channel, the main peaks of Hf 2 and V atoms occurred at around −0.5 eV. In the spin-down channel, in the similar energy region (around −0.5 eV), for V and Hf 2 atoms, such hybridized peaks appeared at the same time. Therefore, the hybridization between the V and Hf 2 atoms that formed strong bonding states at around −0.5 eV. Above the Fermi level, in the spin-up channel, the anti-bonding peak can be found at around 1 eV mainly arise from the Hf 1-d electrons, and in the spin-down channel, no opposite energy states are observed. Moreover, in the spin-up channel, the corresponding bonding-antibonding states led to the formation of an opened band gap, and the Fermi level, exactly, locates between the gap.

For L2_1_ type atomic ordering, the band structures and the DOS have a big difference with XA type atomic ordering. Namely, all the Hf_2_VZ alloys investigated in this work show conventional metallic behaviors without semiconducting-type band gaps at Fermi level in both spin channels. As shown in Figure 3, Figure 4, Figure 5 and Figure 6, both spin-up and spin-down bands are crossed by the Fermi level. 

Moreover, from the obtained total DOS, we calculated the spin polarization (*p*) at Fermi energy of XA and L2_1_ types Hf_2_VZ. The spin polarization *p* (%) that can be defined as the ratio of the difference to sum of the DOS values of spin up and spin down version at the Fermi-level [13,16], represented by mathematical formulation as a percent;

(17) $$p=\frac{n↑(E_{f})-n↓(E_{f})}{n↑(E_{f})+n↓(E_{f})}\times 100\%$$

where the *n*↑ (*E_f_*) and *n*↓ (*E_f_*) stand for the spin-dependent DOS around the Fermi level. The results have been given in Table 3, we can see that the *p* of XA-type Hf_2_VZ are quite high (>88%), even some alloys (such as Hf_2_VAl) have completely spin polarization (100%). High-spin-polarization materials are very useful in spintronic application. However, the L2_1_-type Hf_2_VZ exhibit pretty low spin polarization (<56%). This also implies that the novel spintronic properties (such as half-metallic properties) in XA type structure are disappeared as these alloys form L2_1_ type structure. 

### 3.4. Magnetic and Slater-Pauling Properties of L2_1_ and XA Types Hf_2_VZ

Finally, in this section, we come to discuss the magnetism and Slater-Pauling rule of L2_1_ and XA types Hf_2_VZ (Z = Al, Ga, In, Tl, Si, Ge, Sn, Pb) alloys. The total and atomic magnetic moments of these alloy with XA and L2_1_ atomic ordering are shown in Table 1. Obviously, all these alloys, with either XA or L2_1_ type structures, exhibit FiM properties with the atomic magnetic moments of Hf antiparalled aligned with that of V atoms. Based on Table 1, V carries the largest moment and therefore, V atom makes the most contribution to the total magnetic moment.

For XA-type Hf_2_VZ full-Heusler materials, their total magnetic moments are almost integer values (2 μ_B_/f.u. or 1 μ_B_/f.u.), reflecting their high spin polarization properties. Furthermore, from Table 1, we can see that their total magnetic moments follow the well-known Slater-Pauling rule [37]:

(18) $$M_{t}=Z_{t}-18$$

where the *M_t_* stands for the total magnetic moment of these materials and the *Z_t_* means the number of total of valence electrons in Hf_2_VZ alloys. For L2_1_ type Hf_2_VZ full-Heusler materials, their total magnetic moments are all largely deviated from integer values, indicating the half-metallic properties have been totally destroyed when these alloys are in L2_1_ type structure. For Hf_2_VSi and Hf_2_VGe, both alloys have a very weak moment of 0.24 μ_B_/f.u. and 0.20 μ_B_/f.u., respectively. Therefore, the L2_1_ type Hf_2_VZ full-Heusler alloys do not obey the above-mentioned Slater-Pauling rule.

**Figure 7:**
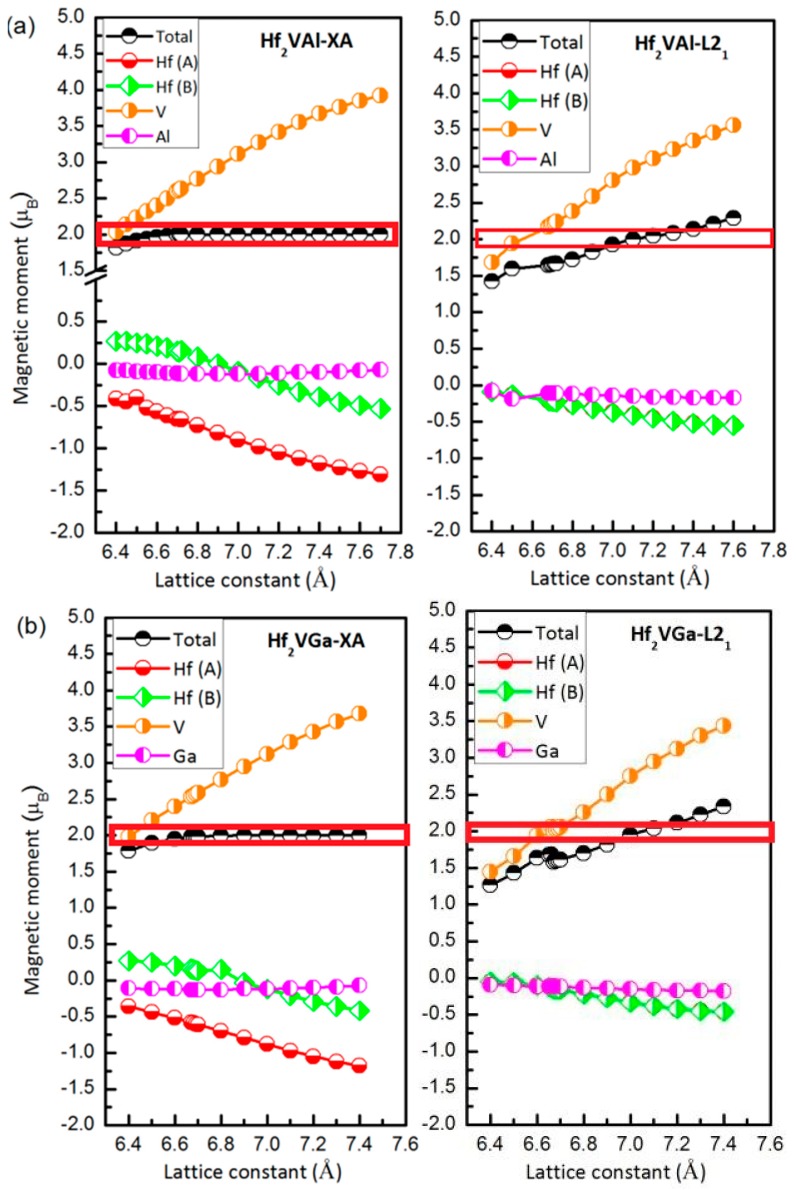
(**a**,**b**) Calculated total and atomic spin magnetic moments of Hf_2_VZ (Z = Al, Ga) alloys as functions of the lattice constant. The red boxes show the high-spin polarization regions.

The magnetism for XA and L2_1_ types full-Heusler alloys Hf_2_VZ (Z = Al, Ga, In, Tl, Si, Sn) alloys at their strained lattice constants has also been examined. As an example, the results of Hf_2_VAl and Hf_2_VGa have been given in Figure 7. According to the Slater-Pauling and generalized electron-filling rules [11,13], the integer values of total magnetic moments which follow the Slater-Pauling rule indicate the half-metallic properties of materials and thus stand for their high spin polarization. From Figure 7, we can see that the high spin polarization properties of XA type Hf_2_VZ (Z = Al, Ga, In, Tl, Si, Sn) alloys are very robust, however, the region with high spin polarization (integer values of total magnetic moments) of L2_1_-type Hf_2_VZ alloys is extraordinarily narrow. For the atomic magnetic moments of XA and L2_1_ types Hf_2_VZ, the values of Hf decreases, whereas the V atom, it increases. 

## 4. Conclusions

To sum up, the atomic occupation of a series of Hf_2_-based full-Heusler alloys was investigated by theoretical first-principle calculations. We observed that all Hf_2_V-based alloys studied in current work are likely to form the L2_1_ structure instead of XA structure. Our results also indicate that the spintronic properties (half-metallic or high spin-polarization properties), and Slater-Pauling behaviors that exist in XA for Hf_2_VZ are fully destroyed in L2_1_. With our study, it is clear that not all full-Heusler alloys X_2_YZ obey the SPR, especially when X are low valence metal elements. 

## Figures and Tables

**Table 1:** The calculated energy difference *ΔE*, lattice constant a, total and atomic spin magnetic moments for Hf_2_VZ alloys with L2_1_ and XA structures, respectively.

Alloy	Structure	*ΔE* (eV/cell)	a (Å)	*M_t_* (μ_B_/f.u.)	*M*_*Hf* 1_ (μ_B_)	*M*_*Hf* 2_ (μ_B_)	*M_V_* (μ_B_)	*M_Z_* (μ_B_)	Stable Structure
Hf_2_VAl	XA	0.64	6.71	2	−0.65	0.14	2.62	−0.12	L2_1_
	L2_1_		6.69	1.65	−0.21	−0.21	2.19	−0.11	
Hf_2_VGa	XA	0.71	6.68	1.99	−0.59	0.15	2.55	−0.13	L2_1_
	L2_1_		6.66	1.68	−0.13	−0.13	2.06	−0.12	
Hf_2_VIn	XA	0.39	6.88	2	−0.75	−0.01	2.88	−0.12	L2_1_
	L2_1_		6.90	1.70	−0.24	−0.24	2.31	−0.13	
Hf_2_VTl	XA	0.32	6.92	1.98	−0.79	−0.1	3.0	−0.12	L2_1_
	L2_1_		6.89	1.61	−0.17	−0.17	2.06	−0.11	
Hf_2_VSi	XA	0.63	6.57	1.02	−0.74	−0.25	2.08	−0.06	L2_1_
	L2_1_		6.56	0.24	−0.03	−0.03	0.31	−0.01	
Hf_2_VGe	XA	0.58	6.64	1.03	−0.83	−0.34	2.26	−0.06	L2_1_
	L2_1_		6.61	0.2	−0.02	−0.02	0.26	−0.01	
Hf_2_VSn	XA	0.18	6.85	1	−1.07	−0.51	2.64	−0.05	L2_1_
	L2_1_		6.82	0.49	−0.06	−0.06	0.63	−0.03	
Hf_2_VPb	XA	0.11	6.94	1	−1.17	−0.63	2.87	−0.04	L2_1_
	L2_1_		6.90	0.32	−0.03	−0.03	0.41	−0.02	

**Table 2:** Calculated elastic constants *C_ij_*, bulk modulus *B*, shear modulus *G*, Young’s modulus *E* (GPa), Pugh’s ratio *B*/*G*, anisotropy factor *A*, and formation energy (eV) for Hf_2_VZ alloys with L2_1_ structure.

Alloy	*C*_11_	*C*_12_	*C*_44_	*B*	*G*	*E*	*B/G*	Formation Energy	Anisotropy Factor
Hf_2_VAl	164.4	117.6	73.0	133.2	46.8	124.6	2.8	−0.54	3.10
Hf_2_VGa	143	104.3	72.9	117.9	43.0	115.10	2.7	−1.19	3.76
Hf_2_VIn	179.8	154.3	49.7	162.8	28.9	82.0	5.6	−0.05	3.89
Hf_2_VTl	110.4	100.1	46.8	103.5	20.5	57.9	5.0	−0.85	9.08
Hf_2_VSi	191	146.9	63.7	161.6	41.6	115.12	3.8	−0.66	2.88
Hf_2_VGe	158.2	123.1	54.1	134.8	34.5	95.4	3.9	−0.41	3.08
Hf_2_VSn	187.5	155.6	42.2	166.2	28.5	81.1	5.8	−0.07	2.64
Hf_2_VPb	109.1	97.9	18.2	101.6	11.4	32.9	8.9	−0.91	3.25

**Table 3:** Calculated valence band maximum (VBM), conduction band minimum (CBM), semiconducting band gaps, spin-flip gap/half-metallic gaps and spin polarization ratio (*p*) of Hf_2_VZ alloys with L2_1_ and XA structures, respectively.

Alloy	Structure	CBM	VBM	Band Gap	Half-Metallic Gap	*p* (%)
Hf_2_VAl	XA	0.23	−0.23	0.46	0.23	100
	L2_1_	-	-	-	-	48
Hf_2_VGa	XA	0.36	−0.23	0.59	0.23	100
	L2_1_	-	-	-	-	52
Hf_2_VIn	XA	0.37	−0.22	0.59	0.22	100
	L2_1_	-	-	-	-	36
Hf_2_VTl	XA	0.47	−0.17	0.64	0.17	100
	L2_1_	-	-	-	-	40
Hf_2_VSi	XA	0.07	−0.28	0.35	0.07	100
	L2_1_	-	-	-	-	39
Hf_2_VGe	XA	-	-	-	-	88
	L2_1_	-	-	-	-	33
Hf_2_VSn	XA	0.04	−0.26	0.30	0.04	100
	L2_1_	-	-	-	-	56
Hf_2_VPb	XA	-	-	-	-	94
	L2_1_	-	-	-	-	36
